# Extraction of moderate oxidation state technetium species between 30 % tri-n-butyl phosphate and H_2_SO_4_/HNO_3_

**DOI:** 10.1007/s10967-015-4122-5

**Published:** 2015-05-10

**Authors:** Maciej Chotkowski

**Affiliations:** Faculty of Chemistry, University of Warsaw, Pasteura 1, 02-093 Warsaw, Poland

**Keywords:** Technetium (III,IV), Solvent extraction, Stability

## Abstract

The extraction of technetium species at oxidation state lower than +7 has been examined in sulfuric and sulfuric/nitric acid solutions using UV–Vis spectroscopy and optically transparent thin layer cell (RVC-OTTLE). Soluble Tc(III), TcO^2+^ and [Tc_2_O_2_]^3+^ species with absorption bands at 420–450, 400, and 502 nm, respectively, were detected as products of pertechnetates electroreduction. The distribution ratios of ^99^Tc with lower than +VII oxidation state ionic species between 4 M H_2_SO_4_ and 30 % TBP/kerosene were found and are significantly lower than for TcO_4_^−^ in the same solution.

## Introduction

^99^Tc is one of the main products of nuclear fission processes and is characterised by a relatively high fission yield of ca. 6 %. Due to a very long half-life (*T*_1/2_ = 2.11 × 10^5^ year) and a very high mobility in the environment, presence of this element strongly complicates nuclear waste storage [1]. Further on due to high ability to co-extract with uranium in TBP (Tri-*n*-butyl phosphate) extraction processes, technetium is present at almost every stage of nuclear fuel reprocessing process [2, 3]. A mixture of technetium species with various oxidation states is formed during the PUREX process. This includes the species with oxidation states of +7 (TcO_4_^−^); +4 or +2 [4] and other unstable soluble species with unclear structure and oxidation state [5].

In general, efficiency of liquid–liquid extraction of technetium species into an organic phase from a weakly acidic aqueous phase is rather poor. This was observed for e.g. Tc(IV) [6] and Tc(II) [7] species and for TcO_4_^−^ [8] also in the absence of the actinides. It was found [8] that the technetium distribution ratio, *D*_Tc_, in ${\text{H}}_2{\text{SO}}_{4{{\text{aq}}^{-}}}$ TBP (45 %)/kerosene system increases with increasing acidity of the aqueous phase: from 4.5 for 0.5 M H_2_SO_4_ to 15.7 for 2 M H_2_SO_4_. The experiments performed by the same author in nitric acid showed an inverse relation in this hydrogen ions concentration range. In the latter case *D*_Tc_ value decreases from 1.36 in 0.5 M HNO_3_ to 0.09 in 4 M HNO_3_. El-Kot [9] reported results of studies on technetium extraction from aqueous solutions with a wide range of nitric acid concentration and using TBP/kerosene as the organic phase. A maximum of *D*_Tc_ with the value slightly higher than unity was observed for ca. 0.6 M of HNO_3_. The extraction behavior of TcO_4_^−^ in the presence of HNO_3_ can be attributed to relatively large amounts of TBP bounded to HNO_3_ that reduce of free TBP available to extract pertechnetates. Higher extraction efficiency of pertechnetates from H_2_SO_4_ as compared to nitric acid solutions was reported also for other than TBP extractants [10–12]. For concentrated sulfuric acid solutions (12 M H_2_SO_4_) is possible to recover technetium from organic into aqueous phase. Such behavior was observed for tetraphenylarsonium chloride as extractant agent [13]. At high acid concentration depressed dissociation of HTcO_4_ is observed which results in decrease of free TcO_4_^−^ ions available to interact with tetraphenylarsonium cations.

For pertechnetates ions the extraction process in acidic media can be expressed by the following reaction [14]:

1 $$ {\text{H}}_{{({\text{aq}})}}^{ + } + {\text{ TcO}}_{{4\;\; ( {\text{aq)}}}}^{ - } + {\text{ 3TBP}}_{{({\text{org}})}} = \, \left[ {{\text{HTcO}}_{ 4} \cdot 3 {\text{TBP}}} \right]_{{({\text{org}})}} $$

Generally, technetium ions with lower than +VII oxidation states are weaker extracted into organic phase than pertechnetates [6, 7, 15] although anionic thiocyanate Tc(V) complexes can be extracted with trioctylphosphine oxide or trioctylamine hydrochloride in cyclohexane or 1,2-dichloroethane as was reported by Boyd [16]. To the best knowledge of the author, no literature date describing the structure of Tc-TBP complexes are available. The only available XAS analysis in sulfuric acid were performed for Tc(VII) and Tc(V) [17–19].

Stability of technetium species at moderate oxidation states is the key factor in understanding technetium behavior in the PUREX process. In my earlier study [20–22] I applied an optically transparent thin layer cell (RVC-OTTLE) in studies of electrochemical behaviour of technetium compounds. We found that stability of non-complexed technetium species in sulfuric acid solutions decreases in the following order TcO_4_^−^ > [Tc_2_O_2_]^3+^ > TcO^2+^ > TcO^+^ (or Tc^3+^). Dimeric structures of Tc(III,IV), [Tc_2_O_2_]^3+^, have been found to be resistant to electrooxidation even at potentials higher than +0.8 V versus SHE [20]. Noteworthy is the fact that all of the above mentioned technetium ionic species can be electrogenerated and characterized spectroscopically using UV or Vis bands [20].

The purpose of this work was to investigate the processes of extraction of selected, electrochemically generated Tc(III), Tc(IV) and Tc(III,IV) species from sulfuric acid medium in the presence and absence of nitric acid.

## Experimental

The selected technetium species, Tc(III), Tc(IV) or Tc(III,IV) were generated electrochemically in a home-made optically transparent thin layer cell with a reticulated vitreous carbon—RVC (thickness 2 mm; 100 ppi porosity, ERG Aerospace Corporation) acting as a working electrode and a platinized platinum gauze serving as a counter electrode [23, 24]. The theory of thin layer spectroelectrochemistry based on RVC was developed by Marassi et al. [25, 26]. A saturated Ag/AgCl electrode was used as a reference electrode but all the potentials in the text are referred to standard hydrogen electrode, SHE. The electroreduction of pertechnetates was carried out in H_2_SO_4_ solutions containing 0.5 mM KTcO_4_ under constant potential conditions, the identity of electroreduction products depends on the applied potential. Hence, Tc(III) species were formed by polarization at 0.35 V (4 M H_2_SO_4_) and 0.01 V (0.5 M H_2_SO_4_) while formation of Tc(IV) was accomplished by polarization at 0.45 V (4 M H_2_SO_4_) and 0.3 V (0.5 M H_2_SO_4_). In the case of 4 M H_2_SO_4_, where pertechnetates reduction is very fast, polarisation time, equal to 15 min, was determined on the basis of UV–Vis spectrometry as the time required to obtain well developed absorption bands indicating generation of specified Tc species with a sufficiently high concentration. A decrease of acidity slows down TcO_4_^−^ reduction, thus, in 0.5 M H_2_SO_4_ polarisation time applied for reduction of pertechnetates was extended to over 3 h. Formation of dimeric structures of Tc(III,IV) was accomplished by electrochemical formation of Tc(III) followed by a slow conversion of the latter into Tc_2_O_2_^3+^ for over 1 week under open circuit potential conditions and at room temperature.

A CHI604 (CH Instruments) electrochemical analyzer and an UV–Vis MultiSpec 1500 (Shimadzu) spectrophotometer were used in the experiments. All the experiments were performed at 293 K. The solutions were prepared using high purity distilled water (Millipore^®^) and high purity chemicals: potassium pertechnetate, K^99^TcO_4_ (Forschungszentrum Dresden-Rossendorf—Institute of Radiopharmacy) H_2_SO_4_ and HNO_3_ (both POCh, Poland) and were deoxygenated with Ar (4 N).

Tc species to be used in the extraction procedures were eluted from the OTTLE cell using 10 ml of sulfuric acid. The extraction experiments were performed by contacting 2 ml of aqueous solutions containing specified Tc species with 2 ml of 30 % TBP in kerosene. After 10 min of intense shaking, 0.1 ml samples were collected from each of the phases and their activities were determined by means of a liquid scintillation counting (uncertainty 5 %) (Perkin Elmer Tri-Carb 2910TR).

Separate experiments were carried out in order to check nitric acid influence on stability of [Tc_2_O_2_]^3+^, TcO^2+^ and Tc(III) in 0.5 M or 4 M H_2_SO_4_. In these experiments, 2 ml portions of sulfuric acid solutions containing electrogenerated Tc species which were eluted from the OTTLE cell by 10 ml of 0.5 M or 4 M H_2_SO_4_ were mixed with 0.1 ml of concentrated HNO_3_. The value of the final concentration of nitric acid, which was equal to 0.67, was selected on the basis of [9] as the concentration with the highest value for extraction between aqueous phase and 30 % TBP/kerosene. After 5, 20 and 35 min from the addition of HNO_3_ into Tc/H_2_SO_4_ a 2.1 ml portion of the Tc/H_2_SO_4_/HNO_3_ mixture was sampled and contacted with 2.1 ml 30 % TBP in kerosene and the extraction procedure was performed. These experiments allowed determination of stability of Tc species in the presence of HNO_3_ as a function of time from the addition of nitric acid. The activities of the phases after extraction were determined by means of liquid scintillation counting, as described in the previous paragraph.

## Results and discussion

**Fig. 1:**
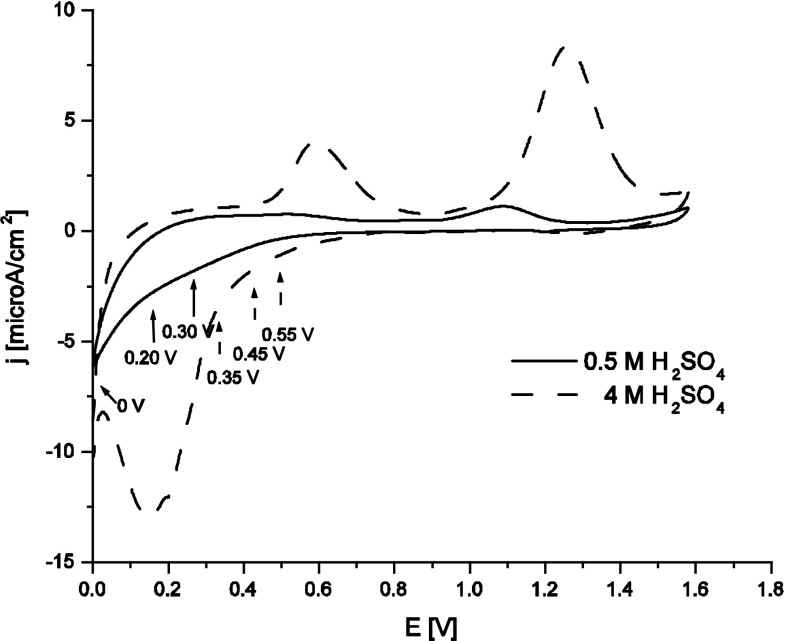
Cyclic voltammograms recorded in 0.5 M H_2_SO_4_ and 4 M H_2_SO_4_ + 0.5 mM KTcO_4_, *v* = 2mVs^−1^, *E*_start_ = 0.8 V, RVC-OTTLE cell, *arrows* indicate potentials applied in chronoamperometric formation of moderate valence state Tc species, see text for details

Figure 1 presents cyclic voltammograms recorded with scan rate of 2 mV/s for a RVC-OTTLE electrode in 0.5 and 4 M H_2_SO_4_ with the addition of 0.5 mM KTcO_4_. The reduction of pertechnetate ions in 0.5 M H_2_SO_4_ leads to a weak increase of cathodic current at potentials lower than 0.4 V.

Simultaneously recorded spectroscopic signal does not reveal significant decrease in concentration of TcO_4_^−^, in agreement with our previous results [20]. Various products of pertechnetates reduction below 0.4 V are proposed in the literature, including TcO_2_·×H_2_O [27], Tc(IV)/Tc(III) species [28] or to Tc(III) which can syn proportionate with Tc(VII) ions to Tc(IV) [29]. The experiments performed in 4 M H_2_SO_4_ showed that the stability of Tc ionic species, Tc(III), Tc(IV) and Tc(III,IV), increases with increasing acidity [20]. Further on, higher currents due to pertechnetates reduction observed in 4 M H_2_SO_4_ indicate that an increase of H_2_SO_4_ concentration facilitates this reaction.

The shape of the anodic section of the voltammogram at potentials of electrooxidation of moderate valence state technetium species to TcO_4_^−^ differs significantly from its cathodic counterpart. Existence of two anodic waves at 0.56 V and 1.0–1.4 V indicate that electrooxidation of moderate valence state technetium to TcO_4_^−^ consists of at least two main steps. Thus, the first anodic wave at 0.56 V can be attributed to electrooxidation of Tc(III) forms to Tc(IV) and possibly to Tc(VII) species. The second anodic wave seen at much higher potentials (1.0–1.4 V) is due to electrooxidation of dimeric structure of Tc(III,IV), [Tc_2_O_2_]^3+^, to pertechnetate ions, the process discussed in detail in our previous paper [20].

**Fig. 2:**
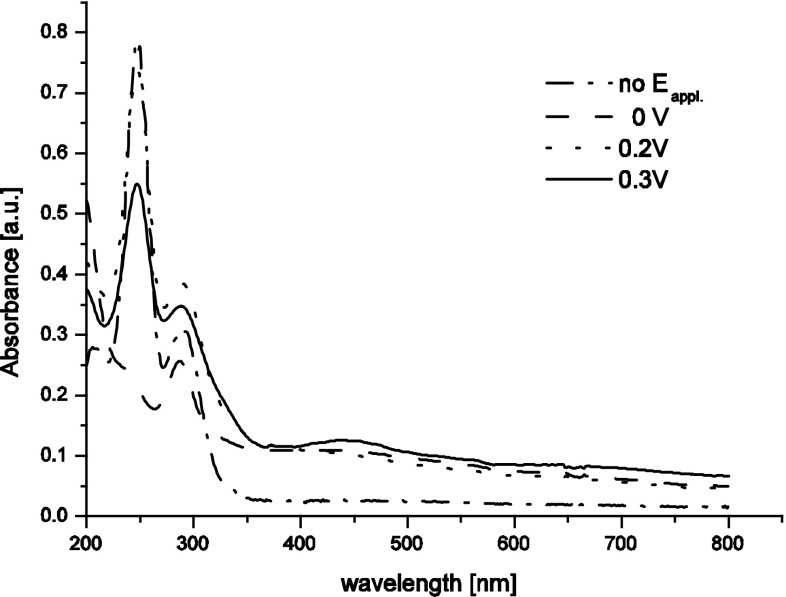
UV–Vis spectra recorded after chronoamperometric polarization of RVC-OTTLE in 0.5 mM KTcO_4_ + 0.5 M H_2_SO_4_ at various potentials indicated in the *plot*

Figure 2 presents UV–Vis spectra for 0.5 M H_2_SO_4_ with the addition of 0.56 mM KTcO_4_ and recorded in OTTLE cell after completing TcO_4_^−^ electroreduction procedure at 0, 0.2 and 0.3 V for 3 h. Presence of aggregates of hydrated Tc(III) and Tc(IV) oxides in 0.5 M H_2_SO_4_ may lead to a significant light scattering and adsorption, as follows from an total increase of the absorbance above 350 nm seen for this electrolyte. An total increase of the absorbance in visible region was observed also during similar experiments led to formation of manganese(III) ions and insoluble manganese dioxide in sulfuric acid solutions [23]. The absorption bands characteristic for pertechnetates (*λ*_max_ = 244 and 288 nm) are observed in 0.5 M H_2_SO_4_ for all applied potentials except the highest one (0.3 V) a poorly developed band with the maximum near 440 nm is seen instead. Thus, the solution contains not only hydrated technetium(III) and (IV) species but also TcO_4_^−^ ions.

**Fig. 3:**
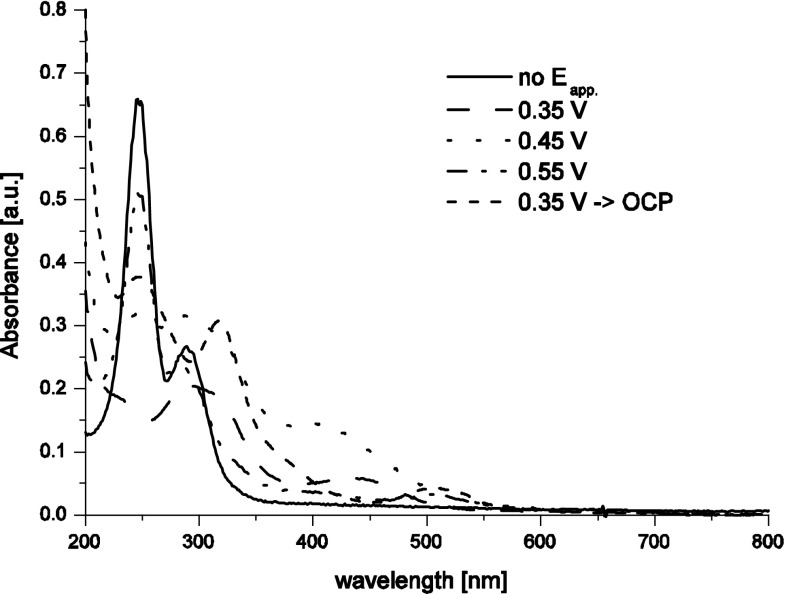
UV–Vis spectra recorded after chronoamperometric polarization of RVC-OTTLE in 0.5 mM KTcO_4_ + 4 M H_2_SO_4_ at various potentials indicated in the *plot*; OCP—open circuit potential

UV–Vis spectra recorded after 15 min of polarization at 0.35, 0.45 and 0.55 V in 4 M H_2_SO_4_ + 0.56 mM KTcO_4_ (Fig. 3) differ significantly from the one recorded for lower H_2_SO_4_ concentration (Fig. 2). In 4 M H_2_SO_4_ technetium(III), (IV) and (III,IV) ionic forms are generated, as indicated by absorption bands observed on the UV–Vis spectrum. The yield of pertechnetates reduction at 0.55 V in 4 M H_2_SO_4_ is very low, hence, dimeric structures of Tc(III,IV), [Tc_2_O_2_]^3+^ (*λ*_max_ = 502 nm) the UV–Vis spectra shown in Fig. 3 reveal existence of non-reduced pertechnetates. Thus, in order to eliminate influence of pertechnetates on extraction of Tc(III,IV), the latter ions were obtained as products of slow transformation/oxidation of TcO^+^/Tc^3+^ ions taking place during a long period a time. This was accomplished by a polarization of the working electrode at 0.35 V followed by keeping the cell under open circuit potential conditions for 1 week. The UV–Vis spectrum recorded after completing such procedure reveals waves with maxima at 502 and 319 nm without signals at 440 nm indicating that Tc(III) ions are slowly oxidized to Tc(III,IV) species. Decrease of the electrode potential to 0.45 V results in generation of technetium(IV) species, TcO^2+^, which are characterized spectroscopically by the band with the maximum near 400 nm. Further decrease of the working electrode potential to 0.35 V leads to generation of Tc(III) soluble species with the absorption band with a maximum near 440 nm. Noteworthy is the fact that for 0.45 V the wave with the maximum near 400 nm is broad and poor shaped which suggest that apart from Tc(IV) also some amounts of Tc(III) can be formed at this polarization potential.

Table 1 collects the results of the extraction experiments for aqueous phases which UV–Vis spectra are shown in Figs. 2 and 3. Generally, for both investigated sulfuric acid concentrations (0.5 and 4 M) the distribution ratio, *D*_Tc_ for all investigated technetium species is lower than for pertechnetates regardless on the nitric acid addition. The TcO_4_^−^ distribution factor obtained in 0.5 M H_2_SO_4_ + HNO_3_ (0.67 M) is equal to This value is in a good agreement with slightly greater than unity value of *D*_Tc_ reported in the literature [9] but is lower than for 0.5 M H_2_SO_4_ and 45 % TBP/kerosene [8]. The highest distribution ratio was calculated for pertechnetates in 4 M H_2_SO_4_ (*D*_Tc_ = 116.16), this value is almost three orders of magnitude higher than for Tc(III) (*D*_Tc_ = 0.06) and Tc(III,IV) (*D*_Tc_ = 0.03) species and about 580 times higher than for Tc(IV) ions. Similarly as in the absence of nitric acid, also for H_2_SO_4_/HNO_3_ solutions the distribution ratio of technetium was the highest for pertechnetates (*D*_Tc_ = 5.47) and decreases to 0.97 for Tc(III), to 0.43 for TcO^2+^ and to 0.05 for [Tc_2_O_2_]^3+^. Obtained results are in line with the literature data [5, 6] which report that distribution ratios for technetium at +II or +IV oxidation state are lower than for pertechnetate ions. The distribution ratio obtained for technetium species generated in 4 M H_2_SO_4_ at potential 0.55 V was higher than for Tc(III) or Tc(IV). It should be bearing in mind that the calculated *D*_Tc_ is an effective value with contributions from all technetium species present in the solution. Thus, apart from Tc(III) and Tc(IV) species also non-reduced pertechnetates may affect significantly obtained values of distribution ratio.

**Table 1:** Distribution ratio of technetium species between aqueous and organic phase electrogenerated at selected potentials

*E*_app._/V	*D*_r_	
	0.5 M H_2_SO_4_	0.5 M H_2_SO_4_ + HNO_3_
0.00	0.09	0.63
0.20	0.77	0.89
0.30	1.26	1.07
No E_app_	1.54	1.25

E_app._/V	4 M H_2_SO_4_	4 M H_2_SO_4_ + HNO_3_
0.35	0.06	0.97
0.45	0.20	0.43
0.55	1.55	1.87
0.35 → OCP	0.03	0.05
No E_app_.	116.16	5.47

Presence of sulfuric acid in aqueous phase results in reduction in the distribution ratio for cationic technetium species in comparison to sulfuric/nitric acid solutions. The sulfate complexes are stronger than nitrate and extract the metal into aqueous phase more effectively from organic medium.

**Fig. 4:**
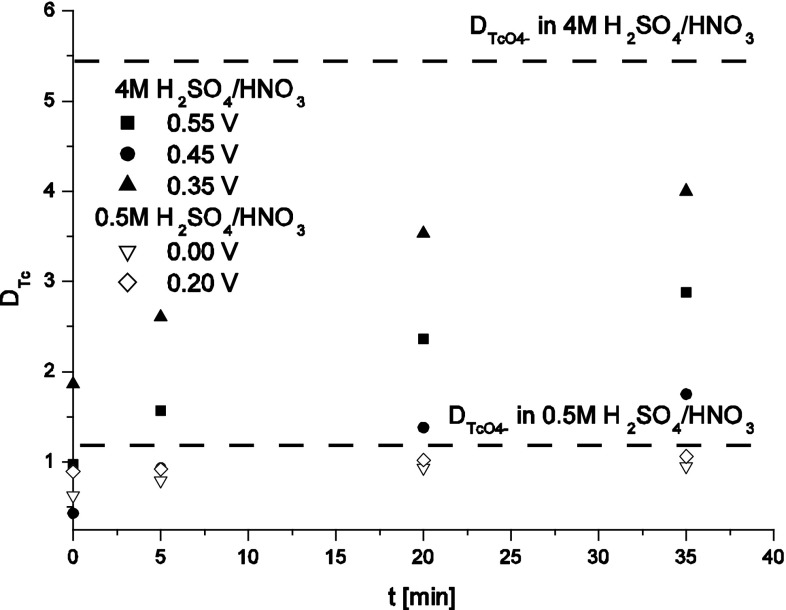
Distribution ratio of technetium between sulfuric or sulfuric/nitric acid solutions and 30 % TBP in kerosene as a function potential applied to formation of moderate valence state Tc species; OCP—open circuit potential

Influence of presence of HNO_3_ on stability of moderate valence state Tc species electrogenerated in 0.5 and 4 M H_2_SO_4_ was tested by means of extraction performed at various time intervals from the addition of HNO_3_ into H_2_SO_4_/Tc solution (Fig. 4. In the case of 4 M H_2_SO_4_/HNO_3_ system the *D*_Tc_ of all investigated Tc species increases with time passed from the addition of HNO_3_ to H_2_SO_4_/Tc solution but even after 35 min from the nitric acid addition its value is lower than for pertechnetates. The highest value of the distribution ratio was observed for the solutions initially containing Tc(III) ions whose presence was confirmed on the basis of electrochemical experiments discussed in [20–22] where it was shown that electrooxidation of Tc(III) takes place at potentials lower than electrooxidation of TcO^2+^ and [Tc_2_O_2_]^3+^. The distribution ratios calculated in 0.5 M H_2_SO_4_/HNO_3_ for reduced technetium species do not differ significantly from the values obtained for pertechnetates and this effect can be attributed to a low yield of formation of Tc(III) and (IV) species electrogeneration and relatively high contribution from unreduced pertechnetates. After 35 min from HNO_3_ addition this factor equals 1.02 and 1.06 for technetium species generated at potentials 0.00 and 0.20 V, respectively, while for pertechnetates *D*_Tc_ is as high as 1.25.

## Conclusions

The electroreduction of pertechnetate ions in acidic media leads to formation of technetium soluble species with +III, +IV and +III,IV oxidation states. The extraction experiments clearly showed that the distribution ratios, *D*_Tc_, of the moderate valence state Tc species between aqueous and organic phase are lower than for pertechnetates. *D*_Tc_ is especially low for dimeric structures of Tc(III,IV). Tc^3+^ ions spectroscopically characterized by a band with the maximum near 440 nm can be easy oxidized to technetium(IV) which exists in the solution as a simple TcO^2+^ ion and dimeric structure of Tc, [Tc_2_O_2_]^3+^.
